# Antimicrobial Resistance as Risk Factor for Recurrent Bacteremia after *Staphylococcus* *aureus*, *Escherichia coli*, or *Klebsiella* spp. Community-Onset Bacteremia

**DOI:** 10.3201/eid3005.231555

**Published:** 2024-05

**Authors:** Salam Abbara, Didier Guillemot, David R.M. Smith, Salma El Oualydy, Maeva Kos, Cécile Poret, Stéphane Breant, Christian Brun-Buisson, Laurence Watier

**Affiliations:** Institut National de la Santé et de la Recherche Médicale, Montigny–Le-Bretonneux, France (S. Abbara, D. Guillemot, C. Brun-Buisson, L. Watier);; Versailles Saint Quentin en Yvelines University, Montigny–Le-Bretonneux (S. Abbara, D. Guillemot, C. Brun-Buisson, L. Watier);; Institut Pasteur, Paris, France (S. Abbara, D. Guillemot, C. Brun-Buisson, L. Watier);; Paris–Cité University, Paris (S. Abbara, D. Guillemot, C. Brun-Buisson, L. Watier);; Paris–Saclay University, Le Kremlin–Bicêtre, France (D. Guillemot);; Assistance Publique–Hôpitaux de Paris, Paris (D. Guillemot, C. Poret, S. Breant);; University of Oxford, Oxford, UK (D.R.M. Smith);; Plateforme de Données de Santé, Paris (S. El Oualydy, M. Kos)

**Keywords:** antimicrobial resistance, bacteremia, drug resistance, recurrence, community-acquired infections, Staphylococcus aureus, Escherichia coli, Klebsiella, bacteria, France

## Abstract

We investigated links between antimicrobial resistance in community-onset bacteremia and 1-year bacteremia recurrence by using the clinical data warehouse of Europe’s largest university hospital group in France. We included adult patients hospitalized with an incident community-onset *Staphylococcus aureus*, *Escherichia coli*, or *Klebsiella* spp. bacteremia during 2017–2019. We assessed risk factors of 1-year recurrence using Fine–Gray regression models. Of the 3,617 patients included, 291 (8.0%) had >1 recurrence episode. Third-generation cephalosporin (3GC)-resistance was significantly associated with increased recurrence risk after incident *Klebsiella* spp. (hazard ratio 3.91 [95% CI 2.32–6.59]) or *E. coli* (hazard ratio 2.35 [95% CI 1.50–3.68]) bacteremia. Methicillin resistance in *S. aureus* bacteremia had no effect on recurrence risk. Although several underlying conditions and infection sources increased recurrence risk, 3GC-resistant *Klebsiella* spp. was associated with the greatest increase. These results demonstrate a new facet to illness induced by 3GC-resistant *Klebsiella spp.* and *E. coli* in the community setting.

Antimicrobial resistance (AMR) is a major global health issue, associated with an estimated 4.95 million deaths worldwide in 2019 (*1*,*2*). Although the effects of AMR on clinical and economic outcomes have been studied extensively, relatively little is known about the effects of AMR on infection recurrence, a significant event that results in substantial illness, death, and healthcare costs (*3*). Recurrence is of particular concern among bacteremia patients, who are often fragile and have underlying conditions, because bacteremia is associated with high rates of death and AMR (*4*). AMR in bacteremia is associated with greater infection severity, higher risk for treatment failure, and longer length of hospital stay, all of which may affect risk for recurrence (*5*–*7*).

Few studies have investigated AMR as a potential risk factor for recurrent bacteremia, and all have been limited to recurrence of infection attributable to the same bacterium that caused initial infection (*8*–*13*). Conversely, the few studies not targeting a specific bacterial species or patient population (e.g., those with underlying conditions) and studying risk factors associated with recurrence within 1 year did not consider AMR as a potential risk factor (*14*–*16*). However, when studying the link between AMR and recurrence, it is important to consider the prolonged microbial imbalance that broad-spectrum antibiotic exposure (i.e., standard bacteremia treatment) can induce on the host microbiome. This imbalance includes ensuing effects on host susceptibility to colonization and infection (*17*) and effects on selection and duration of carriage of antibiotic-resistant bacteria, which, for instance, can exceed 1 year for extended-spectrum β-lactamase (ESBL)–producing Enterobacteriaceae (*18*). AMR in an initial bacteremia episode may thus increase risk for recurrence attributable not only to the same bacterium that caused the initial infection but to any bacterium, whether acquired in the community or hospital. As such, studying bacteremia recurrence across all bacterial species and sources of infection seems clinically relevant. Moreover, it is particularly important to focus on community-onset infections, given the increasing spread of ESBL-producing Enterobacteriaceae in community settings globally (*19*).

In this study, we investigated the effect of AMR in incident community-onset bacteremia on the probability of bacteremia recurrence within 1 year. We restricted incident bacteremia episodes to infections caused by *S. aureus*, *E. coli*, and *Klebsiella* spp., 3 leading pathogens responsible for community-onset bacteremia, and to their leading forms of AMR of major public health concern: methicillin resistance for *S. aureus* and third-generation cephalosporin (3GC) resistance for *E. coli* and *Klebsiella* spp., the major mechanism of which is ESBL production (*20*).

## Material and Methods

### Setting

This observational study used routinely collected data extracted retrospectively from the clinical data warehouse of the Assistance Publique–Hôpitaux de Paris (AP-HP) (https://www.aphp.fr). AP-HP is the largest university hospital group in Europe, with 39 hospitals mainly located in the Greater Paris area and totaling 1.5 million hospitalizations per year (10% of all hospitalizations in France). We focused on 14 AP-HP hospitals with acute care activity, covering ≈22% of all short stays in Île-de-France, the largest region in France. The construction of the database and the included variables have been previously described (*4*). Available data include medical-administrative data describing patient characteristics and hospital stays, as well as microbiological data including infection etiology and antibiotic-susceptibility results. We obtained approval for data collection from the Scientific and Ethical Committee of the Assistance Publique–Hôpitaux de Paris on March 28, 2019. The AP-HP clinical data warehouse initiative ensures that patient information and informed consent regarding the different approved studies are in accordance with European regulations on data protection and authorization number 1980120 from the French Data Protection Authority.

### Study Population

The study population included all patients >18 years of age who were hospitalized with a first clinically important episode of community-onset, monomicrobial bacteremia attributable to *S. aureus*, *E. coli*, or *Klebsiella* spp. in 14 AP-HP university hospitals during January 1, 2017–December 31, 2019. We categorized bacteremia episodes as community-onset if first positive blood culture was collected within 48 hours of admission; otherwise, we categorized the bacteremia as hospital-acquired. We identified incident stays by excluding stays by patients with any history of bacteremia within the previous 12 months, regardless of microbial etiology and location of onset (i.e., whether community-onset or hospital-acquired). We excluded stays ending with death. To avoid including early relapses, we defined recurrence as any clinically important episode of bacteremia (whatever the species, wherever the onset) occurring 7–365 days after hospital discharge from the incident episode (*21*). We identified bacteremia episodes by using microbiologic results (i.e., positive blood cultures) and defined them as previously described (*4*). For statistical analysis, we considered 2 main patient groups: those with recurrence and those without.

### Data Collected

For each patient, data collected were sex, age, and date of death (if applicable). For each incident stay, data collected were dates of admission and discharge; hospital care pathways (e.g., surgery, admission to intensive care unit [ICU], and presence of a septic shock); codes from the International Classification of Diseases, 10th Revision (ICD-10), for underlying conditions; and microbiologic results (e.g., types and dates of microbiologic samples drawn, bacterial species isolated, and antibiotic-susceptibility results). Bacterial antibiotic susceptibilities were determined by the laboratories of participating hospitals by using clinical breakpoints from Comité de l’Antibiogramme de la Société Francaise de Microbiologie–European Committee on Antimicrobial Susceptibility Testing (*22*) and the qualitative susceptibility categories of susceptible, standard dosing regimen (S), susceptible, increased exposure (I), and resistant (R). For antibiotics of interest, we considered strains reported as I to be resistant. When available, empirical therapy data were collected 24 hours before and after the collection date of the first positive blood culture. For each recurrent stay, we collected only dates of hospital admission and discharge and microbiologic results.

### Variables Studied

Patient variables included sex, age group, Charlson comorbidity index (calculated by using comorbidity-associated ICD-10 codes), and comorbidities (i.e., underlying conditions) defined with ICD-10 codes. For each incident stay, other variables considered include patient length of stay (LOS) with bacteremia (the number of days in hospital from the first positive blood culture to the end of the stay), surgery, ICU admission, presence of septic shock, identified bacterial species (*S. aureus*, *E. coli*, or *Klebsiella* spp.), antibiotic susceptibility results, and infection sources (defined according to ICD-10 codes, as previously described) (*4*). Because the effect of resistance may differ according to bacterial species, we considered a 6-class bacteria resistance composite variable (methicillin-susceptible *S. aureus* [MSSA] and methicillin-resistant *S. aureus* [MRSA], 3GC-susceptible and resistant *E. coli*, and 3GC-susceptible and resistant *Klebsiella* spp.) We considered empirical therapy appropriate if >1 antibiotic administered within 24 hours of drawing the first positive blood culture was effective in vitro on the isolated bacteria.

### Statistical Analysis

We described all included patient and hospital stay characteristics according to the presence or absence of recurrence. We also briefly described first recurrent bacteremia and their etiologies. We used Fine–Gray regression models to identify risk factors for recurrence within 1 year after an incident stay, considering death as a competing event. We calculated subdistribution hazard ratios (HRs) by using Gray’s test of the subdistribution function for univariate analyses and Fine–Gray regression models for multivariable analyses. HRs represent the relative change in the instantaneous rate of the occurrence of recurrence in patients who are recurrence-free or who have experienced death, considering patients who have died as nonexposed to recurrence (*23*). The direction of HRs also describes the direction of the effect of covariates on the probability of recurrence occurring over time (incidence) (*23*). We considered variables that had a p value <0.20 in univariate analyses in the multivariable models. We selected variables in the multivariable models by using both backward and forward stepwise methods, and we used a 2-tailed p value <0.05 to define statistical significance. We assessed proportional hazards assumptions in Fine–Gray regressions. We forced the variables age and sex in the multivariable model because they usually are associated with bacterial infections. To confirm results and because the effect of resistance may differ according to bacterial species, we conducted an analysis stratified by bacteria. To assess whether empirical treatment is a confounding factor, we performed an additional analysis considering only patients with information on the adequacy of empirical treatment. For comparability purposes, we estimated a multivariable logistic regression model, considering the same covariates as in the final Fine–Gray regression model. Adjusted odds ratios calculated in the logistic regression model quantify associations between included variables and the odds of bacteremia recurrence, without considering death as a competing event. Finally, to ensure that the type of recurrence, to the same species or to a different species, was not a confounding factor, we estimated a specific Fine–Gray regression model and a multivariable logistic regression model in each of the 2 subgroups, following the same methodology as for the overall sample.

We used HiveQL (https://hive.apache.org), Python 3 (https://www.python.org), PySpark 2.4.3 (https://spark.apache.org/docs/2.4.3), and R 4.0.0 (The R Foundation for Statistical Computing, https://cran.r-project.org) to perform the statistical analyses, and we used the survival R package to compute Fine–Gray regression models (*24*). This study follows the Strengthening the Reporting of Observational Studies in Epidemiology reporting guideline (*25*).

## Results

**Figure 1:**
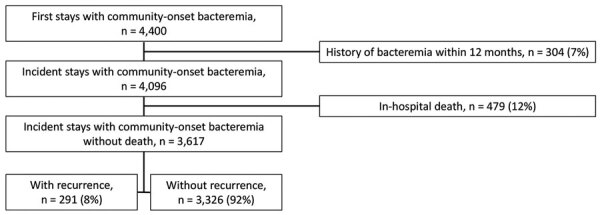
Identification of incident stays of community-onset bacteremia attributable to *Staphylococcus aureus*, *Escherichia coli*, or *Klebsiella* spp., Assistance Publique–Hôpitaux de Paris university hospital group, Paris, France, 2017–2019.

During 2017–2019, we identified 4,400 patients hospitalized with a community-onset bacteremia attributable to *S. aureus*, *E. coli*, or *Klebsiella* spp. We retained their first hospital stay with bacteremia. Of those first stays, 6.9% (n = 304) were excluded because of history of bacteremia within the previous 12 months (Figure 1). Among the remaining 4,096 patients, 11.7% (n = 479) died during their hospital stay and were excluded. In total, we included in the study 3,617 patients with incident hospital stays with community-onset bacteremia attributable to *S. aureus*, *E. coli*, or *Klebsiella* spp. Of those, 8.0% (n = 291) sought treatment for >1 recurrent bacteremia during the following year.

### Descriptive Analyses

#### Incident Stays

Patients with recurrence were more often male than patients without recurrence (56.7% male vs. 47.9% female; p = 0.004) and were more likely to be <80 years of age (81.8% <80 vs. 73.4% >80; age distribution p = 0.0005) (Table 1). Patients with recurrence also had more underlying conditions (27% had a null Charlson comorbidity index vs. 44% of those without recurrence) and were almost twice as likely as their counterparts to have cancer (37.6% vs. 19.5%; p<0.0001), renal disease (22.3% vs. 13.6%; p = 0.002), or liver disease (13.8% vs. 7.5%; p = 0.0007). We observed no statistical difference (p>0.05) between patients with and without recurrence in terms of incident stay characteristics, including LOS with bacteremia (median 7–8 days), rates of surgery, ICU admission, and occurrence of septic shock. However, we observed significant differences regarding the infection source, isolated bacteria, and rates of AMR (p<0.0001 for all). Compared with patients that did not have recurrence, recurrence was more often associated with bacteremia without an identified infection source (24.1% vs. 14.0%) or associated with a digestive (12.1% vs. 9.5%) or device-related infection (7.8% vs. 4.4%) and less often with urinary-source bacteremia (26.2% vs. 35.7%). Moreover, recurrences were more often associated with incident infections attributable to 3GC-resistant *E. coli* (13.1% vs 7.6%), 3GC-susceptible (13.1% vs. 9.3%) or resistant (7.2% vs. 1.9%) *Klebsiella* spp.

**Table 1:** Characteristics of patients and their incident stays with community-onset bacteremia attributable to *Staphylococcus aureus*, *Escherichia coli*, or *Klebsiella* spp., with and without recurrence, Assistance Publique–Hôpitaux de Paris university hospital group, Paris, France, 2017–2019*

Characteristic	With recurrence, n = 291	Without recurrence, n = 3,326	p value
Patients			
Sex			0.004
M	165 (56.7)	1,594 (47.9)	
F	126 (43.3)	1,732 (52.1)	
Age group, y			0.0005
18–35	10 (3.4)	227 (6.8)	
35–50	34 (11.7)	378 (11.4)	
50–65	84 (28.9)	765 (23.0)	
65–80	110 (37.8)	1,070 (32.2)	
>80	53 (18.2)	886 (26.6)	
Charlson comorbidity index†			<0.0001
0	75 (26.6)	1,389 (44.1)	
1–2	109 (38.7)	1,004 (31.9)	
>2	98 (34.7)	7,59 (24.0)	
Underlying conditions†			
Cancer	106 (37.6)	615 (19.5)	<0.0001
Heart failure	35 (12.4)	423 (13.4)	0.61
Diabetes	63 (22.3)	730 (23.2)	0.79
Vascular disease	23 (8.2)	340 (10.8)	0.18
Renal disease	63 (22.3)	430 (13.6)	0.002
Liver disease	39 (13.8)	237 (7.5)	0.0007
Chronic pulmonary disease	12 (4.3)	189 (6.0)	0.24
Dementia	9 (3.2)	178 (5.7)	0.06
Paralysis (hemiplegia or paraplegia)	4 (1.4)	84 (2.7)	0.21
Systemic disease	5 (1.8)	32 (1.0)	0.30
Incident stays			
Length of stay with bacteremia, days			
Median (first quartile–third quartile)	8.0 (4.0–15.5)	7.0 (3.0–15.0)	
Duration, d			0.30
<7	139 (47.8)	1,730 (52.0)	
7–14	74 (25.4)	706 (21.2)	
14–30	50 (17.2)	613 (18.4)	
>30	28 (9.0)	277 (8.3)	
Surgery	37 (12.7)	426 (12.8)	0.97
ICU admission	70 (24.1)	718 (21.6)	0.30
Septic shock†	27 (9.6)	279 (8.9)	0.68
Infection source†			<0.0001
None identified	68 (24.1)	442 (14.0)	
Multiple sites	59 (20.9)	747 (23.7)	
Urinary tract	74 (26.2)	1,125 (35.7)	
Lower respiratory tract	14 (5.0)	190 (6.0)	
Digestive tract	34 (12.1)	300 (9.5)	
Device-related	22 (7.8)	137 (4.4)	
Other	11 (3.9)	211 (6.7)	
Bacteria resistance			<0.0001
MSSA	54 (18.6)	737 (22.2)	
MRSA	4 (1.4)	75 (2.2)	
3GC-susceptible *E. coli*	136 (46.7)	1,889 (56.8)	
3GC-resistant *E. coli*	38 (13.05)	253 (7.6)	
3GC-susceptible *Klebsiella *spp.	38 (13.05)	310 (9.3)	
3GC-resistant *Klebsiella s*pp.	21 (7.2)	62 (1.9)	

*Values are no. (%) except as indicated. p values calculated by using likelihood ratio tests. 3GC, third-generation cephalosporin; ICU, intensive care unit; MRSA, methicillin-resistant *S. aureus*; MSSA, methicillin-susceptible *S. aureus*. †Missing data: 9 stays with recurrence, 174 stays without recurrence.

#### Recurrent Stays

Patients with recurrence had an average of 2.3 (range 2–8 stays) hospital stays with bacteremia over the study period, including their incident stay. First recurrent stays occurred within a median of 80 days (first quartile–third quartile 30.0–175.0 after the incident stay) and were predominantly community-onset (n = 166/291 [57.0%]); median LOS was 11 days (first quartile–third quartile 6.0–19.0 days). The most identified bacteria in first recurrent stays were *E. coli*, *Klebsiella* spp., polymicrobial infection, *S. aureus*, and *Pseudomonas aeruginosa* (Appendix Table 1). In 47.4% of first recurrent episodes (n = 138/291), the same bacterial species was identified as in the incident stay. This rate was higher for *E. coli* (n = 92/174 [53%]) than for *Klebsiella* spp. (n = 25/59 [42%]) or *S. aureus* (n = 21/58 [36%]). In cases of recurrence attributable to the same species, >80% of isolates had the same resistance phenotype as identified in the incident stay (Appendix Table 2).

### Regression Models

Variables not selected for inclusion in the multivariable analysis were heart failure, diabetes, systemic disease, LOS with bacteremia, surgery, ICU admission, and presence of septic shock (Table 2). We did not include Charlson scores in the multivariable model because individual underlying conditions were preferred. In the final model, vascular disease, chronic lung disease, dementia, and presence of paralysis were not retained, and proportional hazards assumptions were validated (p = 0.39).

**Table 2:** Univariable and multivariable analyses of risk factors for bacteremia recurrence at 1 year after an incident stay with community-onset bacteremia attributable to *Staphylococcus aureus*, *Escherichia coli*, or *Klebsiella* spp., Assistance Publique–Hôpitaux de Paris university hospital group, Paris, France, 2017–2019*

Characteristic	Univariable analyses			Multivariable analyses	
	HR (95% CI)	p value		HR (95% CI)	p value
Patients					
Sex; referent male	0.71 (0.57–0.90)	0.004		0.94 (0.73–1.20)	0.59
Age group, y; referent 35–50 y		0.0005			0.075
>18–35	0.51 (0.25–1.03)			0.66 (0.31–1.38)	
50–65	1.23 (0.82–1.83)			1.15 (0.76–1.74)	
65–80	1.14 (0.78–1.69)			1.13 (0.76–1.69)	
>80	0.68 (0.44–1.05)			0.77 (0.49–1.20)	
Charlson comorbidity index; referent 0		<0.0001			
1–2	1.95 (1.46–2.62)				
>2	2.31 (1.71–3.12)				
Underlying conditions					
Cancer	2.36 (1.86–3.00)	<0.0001		2.03 (1.58–2.62)	<0.0001
Heart failure	0.91 (0.64–1.30)	0.61			
Diabetes	0.96 (0.73–1.27)	0.79			
Vascular disease	0.75 (0.49–1.14)	0.18			
Renal disease	1.60 (1.20–2.13)	0.002		1.72 (1.28–2.31)	0.0007
Liver disease	1.89 (1.35–2.65)	0.0007		1.66 (1.17–2.35)	0.007
Chronic pulmonary disease	0.71 (0.40–1.26)	0.24			
Dementia	0.56 (0.29–1.09)	0.06			
Paralysis (hemiplegia / paraplegia)	0.53 (0.20–1.43)	0.21			
Systemic disease	1.70 (0.70–4.12)	0.30			
Incident stays					
Length of stay with bacteremia; referent 7–14 d		0.30			
≤7	0.78 (0.59–1.03)				
14–30	0.79 (0.55–1.13)				
>30	0.97 (0.63–1.50)				
Surgery	1.01 (0.71–1.42)	0.97			
ICU admission	1.15 (0.89–1.51)	0.30			
Septic shock	1.09 (0.73–1.62)	0.68			
Infection source; referent urinary tract		<0.0001			0.0002
None identified	2.25 (1.62–3.13)			2.26 (1.60–3.19)	
Multiple sites	1.19 (0.85–1.68)			1.22 (0.85–1.75)	
Lower respiratory tract	1.12 (0.63–1.98)			1.26 (0.70–2.26)	
Digestive tract	1.69 (1.13–2.54)			1.57 (1.03–2.38)	
Device-related	2.32 (1.44–3.74)			1.93 (1.16–3.23)	
Other	0.80 (0.43–1.51)			0.98 (0.51–1.75)	
Bacteria resistance; referent MSSA		<0.0001			<0.0001
MRSA	0.74 (0.27–2.04)			0.79 (0.29–2.19)	
3GC-susceptible *E. coli*	0.99 (0.72–1.36)			1.16 (0.81–1.66)	
3GC-resistant *E. coli*	1.99 (1.31–3.01)			2.35 (1.50–3.68)	
3GC-susceptible *Klebsiella *spp.	1.64 (1.09–2.49)			1.41 (0.91–2.21)	
3GC-resistant *Klebsiella *spp.	4.11 (2.48–6.81)			3.91 (2.32–6.59)	

*p values calculated by using Gray’s test of the subdistribution function for univariable analyses, and Fine–Gray regression models for multivariable analyses. 3GC, third-generation cephalosporin; HR, subdistribution hazard ratio; ICU, intensive care unit; MRSA, methicillin-resistant *S. aureus*; MSSA, methicillin-susceptible *S. aureus*.

#### Underlying Conditions and Infection Sources

Certain infectious sources were associated with increased recurrence risk within 1 year: absence of an identified infection source (HR 2.26 [95% CI 1.60–3.19]), device-related infection (HR 1.93 [95% CI 1.16–3.23]), and digestive tract infection (HR 1.57 [95% CI 1.03–2.38]). Certain underlying conditions also were identified as associated with increased recurrence risk within 1 year: cancer (HR 2.03 [95% CI 1.58–2.62]), renal disease (HR 1.72 [95% CI 1.28–2.31]), and liver disease (HR 1.66 [95% CI 1.17–2.35]) (Table 2).

#### Antimicrobial Resistance

**Figure 2:**
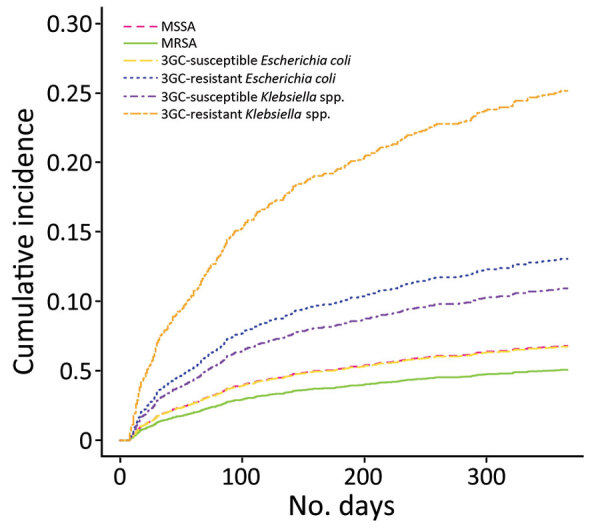
Cumulative incidence function curves showing probability of recurrence over time for each bacteria-resistance pair after community-onset bacteremia attributable to *Staphylococcus aureus*, *Escherichia coli*, or *Klebsiella* spp., Assistance Publique–Hôpitaux de Paris university hospital group, Paris, France, 2017–2019. 3GC, third-generation cephalosporin; ICU, intensive care unit; MRSA, methicillin-resistant *S. aureus*; MSSA, methicillin-susceptible *S. aureus*.

Isolation of MRSA in incident bacteremia episodes did not affect the incidence of recurrence (HR 0.79 [95% CI 0.29–2.19]; referent MSSA). Conversely, isolation of 3GC-resistant *E. coli* (HR 2.02 [95% CI 1.41–2.91]; referent 3GC-susceptible *E. coli*) or 3GC-resistant *Klebsiella* spp. (HR 2.77 [95% CI 1.60–4.79]; referent 3GC-susceptible *Klebsiella* spp.) were associated with an increased risk for recurrence (Table 3). Cumulative incidence function curves of recurrence over time (Figure 2) show the differential effect of bacteria-resistance pairs on risk for recurrence, which was highest for 3GC-resistant *Klebsiella* spp. Those results were similar in an analysis stratified by bacterial species (Table 4) and in the multivariable logistic regression model (Appendix Table 3). A sensitivity analysis considering only stays (36%) with information on empirical treatment showed comparable results, with a higher HR for 3GC-resistant *Klebsiella* spp. and no effect of adequacy of empirical treatment on recurrence risk (Appendix Table 4).

**Table 3:** Subdistribution HRs for relationship between each bacteria-resistance pair and recurrence of bacteremia at 1 year in final multivariable model, by reference, in study of community-onset bacteremia attributable to *Staphylococcus aureus*, *Escherichia coli*, or *Klebsiella* spp., Assistance Publique–Hôpitaux de Paris university hospital group, Paris, France, 2017–2019*

Bacteria resistance	HR (95% CI)		
	Referent MSSA	Referent 3GC-susceptible *E. coli*	Referent 3GC-susceptible *Klebsiella* spp.
MSSA	Referent	0.86 (0.60–1.23)	0.71 (0.45–1.11)
MRSA	0.79 (0.29–2.19)	0.68 (0.25–1.86)	0.56 (0.20–1.59)
3GC-susceptible *E. coli*	1.16 (0.81–1.66)	Referent	0.82 (0.56–1.20)
3GC-resistant *E. coli*	2.35 (1.50–3.68)	2.02 (1.41–2.91)	1.66 (1.04–2.66)
3GC-susceptible *Klebsiella* spp.	1.41 (0.91–2.21)	1.22 (0.83–1.78)	Referent
3GC-resistant *Klebsiella* spp.	3.91 (2.32–6.59)	3.37 (2.10–5.41)	2.77 (1.60–4.79)

*Results are adjusted on all the variables described in Table 2. 3GC, third-generation cephalosporin; HR, subdistribution hazard ratio; MRSA, methicillin-resistant *S. aureus*; MSSA, methicillin-susceptible *S. aureus*.

**Table 4:** Subdistribution HRs for the relationship between bacteria resistance and recurrence of bacteremia at 1 year in analysis stratified by species in study of community-onset bacteremia attributable to *Staphylococcus aureus*, *Escherichia coli*, or *Klebsiella* spp., Assistance Publique–Hôpitaux de Paris university hospital group, Paris, France, 2017–2019*

Bacteria resistance	HR (95% CI)		
	*S. aureus*	*E. coli*	*Klebsiella* spp.
Susceptible	Referent	Referent	Referent
Resistant	0.82 (0.29–2.31)	2.08 (1.44–3.00)	2.41 (1.35–4.30)

*Results were adjusted on all variables included in the multivariable model. Susceptible category included methicillin-susceptible *S. aureus*, and third-generation cephalosporin-susceptible *E. coli* and *Klebsiella* spp. Resistant category included methicillin-resistant *S. aureus*, and third-generation cephalosporin-resistant *E. coli* and *Klebsiella* spp. HR, subdistribution hazard ratio.

Fine–Gray models limited to recurrence to the same or a different species (Appendix Tables 5–7) showed similar HRs for 3GC-resistant *Klebsiella* spp. (referent 3GC-susceptible *Klebsiella* spp.). For 3GC-resistant *E. coli*, the HR was slightly higher for recurrence to the same species (2.47 [95% CI 1.54–3.95]), and slightly lower for recurrence to a different species (1.68 [95% CI 0.94–3.01]; referent 3GC-resistant *E. coli*). Stratified analysis by bacteria found comparable results, except for the HR of the association between 3GC-resistant *Klebsiella* spp. and recurrence to the same species, which was slightly lower (2.32 [95% CI 0.96–5.62]), likely attributable to reduced sample size (Appendix Table 8). Multivariate logistic regression models for recurrence to the same and to different species yielded similar associations (Appendix Table 9).

## Discussion

In this cohort study, we have shown that 3GC resistance in *Klebsiella* spp. or *E. coli* in community-onset bacteremia significantly increases the probability of all-cause bacteremia recurrence within 1 year, whereas identification of MRSA does not affect risk for recurrence. Our results confirm, in community-onset bacteremia, that certain patient underlying conditions (cancer, liver disease, and renal disease) and infection sources (digestive tract, device-related, and no identified infection source) are important risk factors for bacteremia recurrence. Of all identified risk factors, the isolation of 3GC-resistant *Klebsiella* spp. was associated with the greatest increase in the probability of recurrence over time.

Few studies have examined the relationship between AMR and bacteremia recurrence (*8*–*13*). Woudt et al. (*8*) showed an association between the isolation of MRSA or 3GC-resistant Enterobacteriaceae and recurrence of bacteremia attributable to the same species, with crude relative risks of <2. Choi et al. (*9*) found no effect of MRSA on recurrence of *S. aureus* bacteremia over a 7-year study period, after adjustment. One study has focused on the community context and showed crude associations between *E. coli* sequence types 131 or 405, which could be used as a proxy for AMR, and the risk for recurrence (*13*). Our study has shown an effect of 3GC-resistance in community-onset bacteremia attributable to *Klebsiella* spp. or *E. coli* on the probability of recurrence over time, after adjustment for diverse risk factors and while considering death as a competing event. In agreement with Choi et al. (*9*), we found no effect of MRSA on recurrence after adjustment, although our findings are not directly comparable given the community-onset nature of the incident stays and shorter duration of follow-up.

We studied recurrence up to 1 year, accounting for all species and types of bacteremia onset. Although this definition differs from previous works, which focused on recurrence to the same bacterium, we argue that it better captures the potential effect of AMR and ensuing antibiotic exposure on host microbiota and overall susceptibility to infection (*17*). Irrespective of their appropriateness to treat a given bacterium, antibiotics can induce dysbiosis, with repercussions for host immunity, selection of antibiotic-resistant strains, colonization, and infection by antibiotic-susceptible or antibiotic-resistant strains (*17*,*26*–*28*). Given that approximately half the recurrences were attributable to the same species, we conducted sensitivity analyses in the 2 subgroups with recurrence attributable to the same or to a different species. Those analyses showed results consistent with the overall analysis, while suggesting a greater effect of isolating 3GC-resistant *E.coli* during the incident episode on the risk for recurrence attributable to the same species compared with recurrence attributable to a different species. On the other hand, the link between 3GC-resistant *Klebsiella spp.* and the risk for recurrence attributable to the same or a different species was similar. Those results support previous works showing higher rates of illness associated with *Klebsiella spp.* infections compared with *E. coli* infections. Al-Hasan et al. (*29*) showed that isolation of *Klebsiella* spp. was associated with bacteremia recurrence, relative to isolation of *E. coli* and after adjustment. Other work has suggested that patients with ESBL-producing *Klebsiella pneumoniae* bacteremia have higher rates of ICU admission and death compared with patients with ESBL-producing *E. coli* bacteremia (*30*,*31*). Overall, our findings demonstrate a new facet to the disease burden imposed by 3GC resistance in *E. coli* and especially *Klebsiella* spp. infections, the proportions of which are increasing in community settings worldwide (*32*). Our findings support ongoing calls for increased awareness and intervention to limit the spread of antibiotic-resistant *E. coli* and *Klebsiella* spp. in the community. For clinicians in particular, our results highlight the need for increased caution in the follow-up of patients with community-onset bacteremia attributable to 3GC-resistant *Klebsiella* spp. or *E. coli.*

In this study, we have also shown that specific underlying conditions, namely cancer, liver disease, and renal disease, are associated with recurrence, and should thus warrant special attention during patient follow-up. To date, such findings were absent for community-onset bacteremia, and available studies considering certain underlying conditions have shown heterogeneous results (*9*–*12*,*33*,*34*). Furthermore, as previously observed, we underlined that the absence of an infection source or the presence of a digestive or device-related infection source were associated with recurrence (*16*).

Strengths of our study include the large size of our cohort, as well as the richness of the clinical and microbiologic data available, which allowed for the evaluation of potential effects of diverse risk factors. Although the numbers of antibiotic-resistant bacterial isolates in the recurrence group varied, we found statistically significant results for *E. coli* and *Klebsiella* spp. and were able to describe a bacteria-specific effect of resistance on recurrence. Moreover, it is notable that subdistribution HRs calculated with the Fine–Gray regression model were very close to adjusted odds ratios calculated with the multivariable logistic regression model, which supports our results. A close relationship between HRs and ORs values is expected if the event studied has a low probability of occurrence over time (*23*), which is the case in our study, given that the 1-year recurrence rate of bacteremia was 8.0%. This rate was lower than that reported by other studies (9%–12%), which could be explained by the selection of community-onset incident stays (*14**–**16*) or by the fact that recurrent stays were only identified among AP-HP hospitals.

Despite the size of our cohort, this study is not population-based, given that it covers approximately one quarter of all acute care inpatients in Île-de-France. Moreover, it included patients hospitalized in university hospitals, who may have more underlying conditions and exposures to care, affecting the risk for recurrence. To minimize this bias, we adjusted our results on most previously identified risk factors of recurrence, which could be related to patients or their hospital stay and infection characteristics (*14*–*16*). Because data on exposure to care was only available among AP-HP hospitals, we could not study this risk factor and used a commonly accepted definition of community-onset infections as occurring within the first 48 hours of admission (*15*,*16*). Moreover, information on empirical treatment, which could affect recurrence risk, was only available for one third of included cases because of the multiplicity of drug prescription software platforms used across included hospitals (*14*,*16*). Despite this limitation, a sensitivity analysis on patients with information on their empirical treatment showed similar results to the main analysis, thereby supporting our findings.

In conclusion, we have shown that resistance to 3GCs in *Klebsiella* spp. and *E. coli* during incident community-onset bacteremia significantly increases bacteremia recurrence risk over time. This risk was highest for 3GC-resistant *Klebsiella* spp., for which increasing community dissemination represents an urgent public health problem. These findings reveal an important facet to the disease and death induced by antimicrobial-resistant Enterobacteriaceae and inform a need for careful follow-up of patients recovering from bacteremia caused by these bacteria, as well as and a need for interventions to limit their further spread in the community.

Appendix Additional information about antimicrobial resistance as risk factor for recurrent bacteremia after *Staphylococcus aureus*, *Escherichia coli*, or *Klebsiella* spp. community-onset bacteremia.
