# Supplementary material for: Antimicrobial Resistance as Risk Factor for Recurrent Bacteremia after Staphylococcus aureus, Escherichia coli, or Klebsiella spp. Community-Onset Bacteremia
Source: Emerg Infect Dis. 2024 May;30(5):974–83. doi: 10.3201/eid3005.231555 (PMC11060452; doi:10.3201/eid3005.231555)
Supplement: Appendix — Additional information about antimicrobial resistance as risk factor for recurrent bacteremia after Staphylococcus aureus, Escherichia coli, or Klebsiella spp. community-onset bacteremia. [file 23-1555-Techapp-s1.pdf]

Article DOI: https://doi.org/10.3201/eid3005.231555

Antimicrobial Resistance as Risk Factor for Recurrent Bacteremia after *Staphylococcus aureus*, *Escherichia coli*, or *Klebsiella* spp. Community-Onset Bacteremia

Appendix

Appendix Table 1. Most frequently isolated bacteria in first recurrent episodes, stratified by the bacterial species isolated in incident episodes.

Bacteria, first recurrent episodes, no.	Incident episode due to *E. coli* (no. = 174)	Incident episode due to *Klebsiella* spp. (no. = 59)	Incident episode due to *S. aureus* (no. = 58)
E. coli	92	9	8
Polymicrobial	25	8	6
Klebsiella spp.	14	25	3
S. aureus	6	3	21
P. aeruginosa	5	5	4
Others	32	9	16

Appendix Table 2. Concordance between the bacterial resistance phenotype of incident and first recurrent episodes, among recurrences due to the same species as the incident episode.

Bacteria-resistance	Same phenotype, no./NO.
E. coli	
3GC-S	63/68
3GC-R	23/24
Klebsiella spp.	
3GC-S	14/15
3GC-R	8/10
S. aureus	
Methicillin-S	19/20
Methicillin-R	1/1

3GC-S / -R: susceptible / resistant to 3rd-generation cephalosporins; methicillin-S / -R: methicillin- susceptible / -resistant.

Appendix Table 3. Multivariable logistic regression models of risk factors for bacteremia recurrence at 1 y, following an incident stay with community-onset bacteremia due to *S. aureus, E. coli,* or *Klebsiella* spp., 2017–2019

	Recurrence to any species	
Characteristic	aOR [95CI]	P-value
Patients		
Sex (ref = male)	0.93 [0.71 – 1.21]	0.56
Age, years (ref = [35–50])		0.06
[18–35]	0.62 [0.27 – 1.30]	
[50–65]	1.14 [0.74 – 1.80]	
[65–80]	1.12 [0.73 – 1.75]	
>80	0.76 [0.47 – 1.23]	
Comorbidities		
Cancer (ref = absence)	2.19 [1.66 – 2.88]	<0.0001
Renal disease (ref = absence)	1.78 [1.28 – 2.45]	0.0005
Liver disease (ref = absence)	1.80 [1.21 – 2.62]	0.006
Incident stays		
Infection source (ref = urinary tract)		0.0003
None identified	2.41 [1.66 – 3.49]	
Multiple sites	1.24 [0.84 – 1.81]	

Characteristic	Recurrence to any species aOR [95CI]	P-value
Lower respiratory tract	1.25 [0.65 – 2.24]	
Digestive tract	1.61 [1.02 – 2.49]	
Device-related	1.97 [1.10 – 3.41]	
Other	0.98 [0.47 – 1.89]	
Bacteria-resistance (ref = MSSA)		<0.0001
MRSA	0.77 [0.23 – 1.99]	
3GC-S *E. coli*	1.15 [0.79 – 1.68]	
3GC-R *E. coli*	2.48 [1.53 – 4.01]	
3GC-S *Klebsiella* spp.	1.43 [0.88 – 2.28]	
3GC-R *Klebsiella* spp.	4.70 [2.52 – 8.58]	

P-values were calculated using multivariable logistic regression models. Abbreviations: aOR: adjusted odds ratio; 3GC-S / 3GC-R: 3rd-generation cephalosporin-susceptible / -resistant; ICU: intensive care unit; MSSA / MRSA: methicillin-susceptible / -resistant *S. aureus*.

Appendix Table 4. Descriptive and Fine-Gray multivariable analyses of risk factors for bacteremia recurrence at 1 y, following an incident stay with community-onset bacteremia due to *S. aureus, E. coli,* or *Klebsiella* spp. in the subsample of incident stays including information on the adequacy of empirical treatment, 2017–2019.

	Description of the population, N (%)		Multivariable analyses	
Characteristic	With recurrence N = 117	Without recurrence N = 1175	HR [95CI]	P-value
Patients				
Sex, no. (%)				0.45
Male	67 (57.3)	522 (44.4)	1	
Female	50 (42.7)	653 (55.6)	0.86 [0.58 - 1.27]	
Age, years, no. (%)				0.006
[18–35]	1 (0.9)	80 (6.8)	0.17 [0.02 - 1.32]	
[35–50]	12 (10.3)	130 (11.1)	1	
[50–65]	39 (33.3)	254 (21.6)	1.58 [0.82 - 3.05]	
[65–80]	44 (37.6)	392 (33.4)	1.10 [0.57 - 2.12]	
>80	21 (17.9)	319 (27.1)	0.75 [0.36 - 1.55]	
Comorbidities*, no. (%)				
Cancer	40 (34.5)	218 (19)	1.73 [1.14 - 2.62]	0.01
Renal disease	25 (21.6)	171 (14.9)	1.63 [1.02 - 2.59]	0.05
Liver disease	16 (13.8)	82 (7.2)	1.62 [0.93 - 2.82]	0.11
Incident stays				
Infection source*, no. (%)				0.005
None identified	25 (21.6)	127 (11.1)	3.04 [1.75 - 5.29]	
Multiple sites	27 (23.3)	262 (22.9)	1.72 [0.99 - 2.97]	
Lower respiratory tract	6 (5.2)	47 (4.1)	2.38 [0.96 - 5.92]	
Urinary tract	30 (25.9)	481 (42)	1	
Digestive tract	11 (9.5)	104 (9.1)	1.60 [0.79 - 3.25]	
Device-related	12 (10.3)	52 (4.5)	3.20 [1.54 - 6.69]	
Other	5 (4.3)	73 (6.4)	1.75 [0.63 - 4.84]	
Bacteria-resistance, no. (%)				0.003
MSSA	17 (14.5)	205 (17.4)	1	
MRSA	2 (1.7)	24 (2)	0.93 [0.21 - 4.09]	
3GC-S *E. coli*	54 (46.2)	705 (60)	1.44 [0.79 - 2.63]	
3GC-R *E. coli*	20 (17.1)	113 (9.6)	2.84 [1.33 - 6.09]	
3GC-S *Klebsiella* spp.	13 (11.1)	105 (8.9)	1.41 [0.65 - 3.05]	
3GC-R *Klebsiella* spp.	11 (9.4)	23 (2)	6.57 [2.69 - 16.06]	
Adequacy of empiric treatment, no. (%)				0.51
Appropriate	95 (81.2)	1072 (91.2)	1	
Inappropriate	22 (18.8)	103 (8.8)	1.22 [0.68 - 2.17]	

Abbreviations: 3GC-S / 3GC-R: susceptible / resistant to 3rd-generation cephalosporins; HR: subdistribution hazard ratios; ICU: intensive care unit; MSSA / MRSA: methicillin-susceptible / resistant *S. aureus*. *Missing data: 1 stay with recurrence, 29 stays without recurrence.

Appendix Table 5. Univariable and multivariable analyses of risk factors for bacteremia recurrence to the same species at 1 y, following an incident stay with community-onset bacteremia due to *S. aureus, E. coli,* or *Klebsiella* spp., 2017–2019.

	Univariable analyses		Multivariable analyses	
Characteristic	HR [95CI]	P-value	HR [95CI]	P-value
Patients				
Sex (ref = male)	0.75 [0.54 – 1.06]	0.1	0.90 [0.63 – 1.29]	0.56
Age (ref = [35–50])		0.07		0.14
≥18–35]	0.45 [0.15 – 1.36]		0.44 [0.13 – 1.53]	
[50–65]	1.29 [0.71 – 1.33]		1.30 [0.71 – 2.37]	
[65–80]	1.23 [0.69 – 1.18]		1.19 [0.66 – 2.13]	
>80	0.81 [0.43 – 1.51]		0.82 [0.43 – 1.57]	
Charlson comorbidity index (ref = 0)		0.02		
1–2	1.65 [1.11 – 2.46]			
>2	1.60 [1.04 – 2.47]			
Comorbidities				
Cancer	1.52 [1.04 – 2.22]	0.03		
Heart failure	0.92 [0.56 – 1.53]	0.76		
Diabetes	1.03 [0.69 – 1.53]	0.90		
Vascular disease	0.88 [0.50 – 1.56]	0.66		
Renal disease	2.16 [1.47 – 3.17]	<0.0001	2.04 [1.37 – 3.03]	0.0009
Liver disease	1.94 [1.19 – 3.14]	0.008	1.70 [1.03 – 2.80]	0.05
Chronic pulmonary disease	0.36 [0.12 – 1.13]	0.08		
Dementia	0.51 [0.19 – 1.39]	0.19		
Paralysis (hemiplegia / paraplegia)	0.55 [0.14 – 2.21]	0.40		
Systemic disease	0.72 [0.10 – 5.17]	0.75		
Incident stays				
Length of stay with bacteremia (ref = [7–14])		0.30		
≤7	0.74 [0.50 – 1.10]			
[14–30]	0.64 [0.38 – 1.10]			
>30	0.74 [0.38 – 1.45]			
Surgery	1.15 [0.71 – 1.84]	0.58		
ICU admission	1.17 [0.80 – 1.73]	0.42		
Septic shock	1.09 [0.62 – 1.93]	0.77		
Infection source (ref = urinary tract)		0.20		0.17
None identified	1.49 [0.92 – 2.39]		1.87 [1.14 – 3.06]	
Multiple sites	1.88 [0.55 – 1.42]		1.06 [0.64 – 1.74]	
Lower respiratory tract	0.65 [0.26 – 1.64]		0.84 [0.33 – 2.15]	
Digestive tract	1.37 [1.78 – 2.38]		1.48 [0.83 – 2.62]	
Device-related	1.41 [1.67 – 2.99]		1.90 [0.86 – 4.20]	
Other	0.70 [0.30 – 1.63]		1.10 [0.45 – 2.71]	
Bacteria-resistance (ref = MSSA)		<0.0001		<0.0001
MRSA	0.49 [0.07 – 3.68]		0.53 [0.07 – 3.95]	
3GC-S *E. coli*	1.33 [0.81 – 2.19]		1.68 [0.95 – 2.95]	
3GC-R *E. coli*	3.41 [1.89 – 6.18]		4.13 [2.14 – 7.98]	
3GC-S *Klebsiella spp*	1.77 [0.90 – 3.45]		1.85 [0.91 – 3.73]	
3GC-R *Klebsiella spp*	5.55 [2.60 – 11.86]		5.62 [2.54 – 12.41]	

P-values were calculated using Gray's test of the subdistribution function for univariable analyses, and Fine-Gray regression models for multivariable analyses. Abbreviations: 3GC-S / 3GC-R: 3rd-generation cephalosporin-susceptible / -resistant; HR: subdistribution hazard ratios; ICU: intensive care unit; MSSA / MRSA: methicillin-susceptible / -resistant *S. aureus*.

Appendix Table 6. Univariable and multivariable analyses of risk factors for bacteremia recurrence to a different species at 1 y, following an incident stay with community-onset bacteremia due to *S. aureus, E. coli,* or *Klebsiella* spp., 2017–2019.

	Univariable analyses		Multivariable analyses	
Characteristic	HR [95CI]	P-value	HR [95CI]	P-value
Patients				
Sex (ref = male)	0.67 [0.48 – 0.92]	0.01	0.92 [0.65 – 1.30]	0.63
Age (ref = [35–50])		0.01		0.46
≥18–35]	0.54 [0.21 – 1.34]		0.90 [0.35 – 2.29]	
[50–65]	1.17 [0.69 – 2.01]		1.09 [0.61 – 1.92]	
[65–80]	1.08 [0.64 – 1.81]		1.16 [0.67 – 2.03]	
>80	0.57 [0.31 – 1.03]		0.75 [0.40 – 1.42]	
Charlson comorbidity index (ref = 0)		<0.0001		
1–2	2.45 [1.58 – 3.80]			
>2	3.40 [2.20 – 5.25]			
Comorbidities				
Cancer	3.56 [2.57 – 4.92]	<0.0001	2.86 [2.03 – 4.03]	<0.0001
Heart failure	0.91 [0.55 – 1.48]	0.69		
Diabetes	0.90 [0.60 – 1.33]	0.59		

Characteristic	Univariable analyses HR [95CI]	P-value	Multivariable analyses HR [95CI]	P-value
Vascular disease	0.62 [0.32 – 1.17]	0.14		
Renal disease	1.17 [0.75 – 1.83]	0.48		
Liver disease	1.92 [1.20 – 3.08]	0.007	1.73 [1.06 – 2.81]	0.04
Chronic pulmonary disease	1.03 [0.52 – 2.01]	0.94		
Dementia	0.60 [0.25 – 1.46]	0.26		
Paralysis (hemiplegia / paraplegia)	0.52 [0.13 – 2.08]	0.35		
Systemic disease	2.65 [0.98 – 7.17]	0.05	3.26 [1.18 – 8.98]	0.05
Incident stays				
Length of stay with bacteremia (ref = [7–14])		0.50		
≤7	0.81 [0.54 – 1.21]			
[14–30]	0.93 [0.57 – 1.52]			
>30	1.20 [0.68 – 2.14]			
Surgery	0.88 [0.53 – 1.45]	0.61		
ICU admission	1.15 [0.79 – 1.67]	0.48		
Septic shock	1.09 [0.63 – 1.89]	0.77		
Infection source (ref = urinary tract)		<0.0001		0.0007
None identified	3.60 [2.23 – 5.82]		3.04 [1.85 – 5.00]	
Multiple sites	1.71 [1.03 – 2.84]		1.49 [0.87 – 2.55]	
Lower respiratory tract	1.89 [0.89 – 3.99]		1.90 [0.88 – 4.11]	
Digestive tract	2.27 [1.24 – 4.15]		1.83 [0.99 – 3.38]	
Device-related	3.96 [2.08 – 7.52]		2.40 [1.19 – 4.82]	
Other	0.96 [0.37 – 2.48]		0.99 [0.37 – 2.66]	
Bacteria-resistance (ref = MSSA)		<0.0001		0.01
MRSA	0.88 [0.27 – 2.86]		0.93 [0.28 – 3.05]	
3GC-S *E. coli*	0.79 [0.52 – 1.19]		0.91 [0.57 – 1.44]	
3GC-R *E. coli*	1.19 [0.64 – 2.22]		1.52 [0.79 – 2.93]	
3GC-S *Klebsiella spp*	1.59 [0.94 – 2.71]		1.22 [0.68 – 2.17]	
3GC-R *Klebsiella spp*	3.63 [1.84 – 7.17]		3.51 [1.75 – 7.06]	

P-values were calculated using Gray's test of the subdistribution function for univariable analyses, and Fine-Gray regression models for multivariable analyses. Abbreviations: 3GC-S / 3GC-R: 3rd-generation cephalosporin-susceptible / -resistant; HR: subdistribution hazard ratios; ICU: intensive care unit; MSSA / MRSA: methicillin-susceptible / -resistant *S. aureus*.

Appendix Table 7. Subdistribution hazard ratios and 95% confidence intervals for the relationship between each bacteria-resistance pair and recurrence of bacteremia at 1 y in the final multivariable model as a function of the recurrence type, by reference*, 2017–2019

Bacteria-resistance	HR [ref = MSSA]	HR [ref = 3GC-S *E. coli*]	HR [ref = 3GC-S *Klebsiella* spp.]
Recurrence to any species			
MSSA	1	0.86 [0.60 – 1.23]	0.71 [0.45 – 1.11]
MRSA	0.79 [0.29 – 2.19]	0.68 [0.25 – 1.86]	0.56 [0.20 – 1.59]
3GC-S *E. coli*	1.16 [0.81 – 1.66]	1	0.82 [0.56 – 1.20]
3GC-R *E. coli*	2.35 [1.50 – 3.68]	2.02 [1.41 – 2.91]	1.66 [1.04 – 2.66]
3GC-S *Klebsiella* spp.	1.41 [0.91 – 2.21]	1.22 [0.83 – 1.78]	1
3GC-R *Klebsiella* spp.	3.91 [2.32 – 6.59]	3.37 [2.10 – 5.41]	2.77 [1.60 – 4.79]
Recurrence to the same species			
MSSA	1	0.60 [0.34 – 1.05]	0.54 [0.27 – 1.10]
MRSA	0.53 [0.07 – 3.95]	0.31 [0.04 – 2.31]	0.29 [0.04 – 2.19]
3GC-S *E. coli*	1.68 [0.95 – 2.95]	1	0.91 [0.51 – 1.61]
3GC-R *E. coli*	4.13 [2.14 – 7.98]	2.47 [1.54 – 3.95]	2.24 [1.15 – 4.35]
3GC-S *Klebsiella* spp.	1.85 [0.91 – 3.73]	1.10 [0.62 – 1.95]	1
3GC-R *Klebsiella* spp.	5.62 [2.54 – 12.41]	3.35 [1.69 – 6.63]	3.05 [1.35 – 6.86]
Recurrence to a different species			
MSSA	1	1.10 [0.70 – 1.75]	0.82 [0.46 – 1.47]
MRSA	0.93 [0.28 – 3.05]	1.03 [0.32 – 3.33]	0.77 [0.22 – 2.62]
3GC-S *E. coli*	0.91 [0.57 – 1.44]	1	0.74 [0.45 – 1.24]
3GC-R *E. coli*	1.52 [0.79 – 2.93]	1.68 [0.94 – 3.01]	1.25 [0.63 – 2.50]
3GC-S *Klebsiella* spp.	1.22 [0.68 – 2.17]	1.34 [0.81 – 2.24]	1
3GC-R *Klebsiella* spp.	3.51 [1.75 – 7.06]	3.88 [2.01 – 7.48]	2.88 [1.37 – 6.07]

* Results are adjusted on all the variables described in Table 2, Appendix Table 5 and 6 for recurrence to any species, recurrence to the same species, and recurrence to a different species, respectively. Abbreviations: 3GC-S / 3GC-R: susceptible / resistant to 3rd-generation cephalosporins; HR: subdistribution hazard ratios; MSSA / MRSA: methicillin-susceptible / -resistant *S. aureus*; OR: odds ratios.

Appendix Table 8. Count of bacterial isolates, and subdistribution hazard ratios with 95% confidence intervals for the relationship between antimicrobial resistance and recurrence of bacteremia at 1 y as a function of the recurrence type, in an analysis stratified by species*, 2017–2019

	S. aureus		*E. coli*		*Klebsiella* spp.	
Characteristic	No. with / without recurrence	HRs	No. with / without recurrence	HRs	No. with / without recurrence	HRs
Recurrence to any species						
Susceptible	54/737	1	136/1889	1	38/310	1
Resistant	4/75	0.82 [0.29 – 2.31]	38/253	2.08 [1.44 - 3.00]	21/62	2.41 [1.35 - 4.30]
Recurrence to the same species						
Susceptible	20/737	1	68/1889	1	15/310	1
Resistant	1/75	0.49 [0.06 – 3.80]	24/253	2.59 [1.61 - 4.17]	10/62	2.32 [0.96 - 5.62]
Recurrence to a different species						
Susceptible	34/737	1	68/1889	1	23/310	1
Resistant	3/75	0.99 [0.30 – 3.29]	14/253	1.67 [0.93 - 3.01]	11/62	2.93 [1.35 - 6.36]

The susceptible category included methicillin-susceptible *S. aureus*, and 3rd-generation cephalosporin susceptible *E. coli* and *Klebsiella* spp. The resistant category included methicillin-resistant *S. aureus*, and 3rd-generation cephalosporin-resistant *E. coli* and *Klebsiella* spp. *For each bacteria, results were adjusted on all variables included in the multivariable model of the recurrence group.

Appendix Table 9. Multivariable logistic regression models of risk factors for bacteremia recurrence at 1 y, following an incident stay with community-onset bacteremia due to *S. aureus, E. coli,* or *Klebsiella* spp., 2017–2019, by recurrence type

	Recurrence to the same species		Recurrence to a different species	
Characteristic	aOR [95CI]	P-value	aOR [95CI]	P-value
Patients				
Sex (ref = male)	0.90 [0.62 - 1.30]	0.56	0.92 [0.64 - 1.31]	0.64
Age, years (ref = [35–50])		0.14		0.47
[18–35]	0.42 [0.10 - 1.31]		0.86 [0.30 - 2.16]	
[50–65]	1.28 [0.70 - 2.46]		1.09 [0.61 - 2.04]	
[65–80]	1.18 [0.66 - 2.22]		1.16 [0.66 - 2.14]	
>80	0.81 [0.42 - 1.62]		0.75 [0.39 - 1.47]	
Comorbidities				
Cancer	-	-	3.02 [2.11 - 4.32]	<0.0001
Renal disease	2.08 [1.35 - 3.12]	0.0006	-	-
Liver disease	1.80 [1.04 - 2.99]	0.04	1.77 [1.03 - 2.93]	0.04
Systemic disease		-	3.57 [1.01 - 9.73]	0.048
Incident stays				
Infection source (ref = urinary tract)		0.17		0.0006
None identified	1.91 [1.13 - 3.16]		3.24 [1.93 - 5.48]	
Multiple sites	1.06 [0.63- 1.76]		1.52 [0.88 - 2.65]	
Lower respiratory tract	0.83 [0.28 - 1.99]		1.93 [0.82 - 4.12]	
Digestive tract	1.53 [0.83 - 2.73]		1.84 [0.95 - 3.44]	
Device-related	1.86 [0.76 - 4.07]		2.48 [1.16 - 5.11]	
Other	1.10 [0.40 - 2.61]		1.01 [0.33 - 2.55]	
Bacteria-resistance (ref = MSSA)		<0.0001		0.01
MRSA	0.53 [0.03 - 2.63]		0.92 [0.21 - 2.71]	
3GC-S *E. coli*	1.66 [0.95 - 3.03]		0.90 [0.56 - 1.47]	
3GC-R *E. coli*	4.24 [2.15 - 8.45]		1.55 [0.76 - 3.02]	
3GC-S *Klebsiella* spp.	1.86 [0.89 - 3.82]		1.22 [0.65 - 2.21]	
3GC-R *Klebsiella* spp.	6.17 [2.55 - 14.21]		3.99 [1.76 - 8.54]	

P-values were calculated using multivariable logistic regression models. Abbreviations: aOR: adjusted odds ratio; 3GC-S / 3GC-R: 3rd-generation cephalosporin-susceptible / -resistant; ICU: intensive care unit; MSSA / MRSA: methicillin-susceptible / -resistant *S. aureus*.
